# Personality of organizational social media accounts and its relationship with characteristics of their photos: analyses of startups’ Instagram photos

**DOI:** 10.1186/s40359-024-01709-6

**Published:** 2024-04-25

**Authors:** Yunhwan Kim

**Affiliations:** College of General Education, Kookmin University, 801 Bugak Hall, 77 Jeongneung-ro, Seongbuk-gu, 02707 Seoul, South Korea

**Keywords:** Startup, Instagram, Big five personality, Social media, Visual communication

## Abstract

**Background:**

Organizational accounts of social networking sites (SNSs) are similar to individual accounts in terms of their online behaviors. Thus, they can be investigated from the perspective of personality, as individual accounts have been in the literature. Focusing on startups’ Instagram accounts, this study aimed to investigate the characteristics of Big Five personality traits and the relationships between the traits and the characteristics of photos in organizational SNS accounts.

**Methods:**

The personality traits of 108 startups’ accounts were assessed with an online artificial intelligence service, and a correspondence analysis was performed to identify the key dimensions where the account were distributed by their personality. Photo features were extracted at the content and pixel levels, and correlational analyses between personality traits and photo features were conducted. Moreover, predictive analyses were performed using random forest regression models.

**Results:**

The results indicated that personality of the accounts had high openness, agreeableness, and conscientiousness and moderate extraversion and neuroticism. In addition, the two dimensions of high vs. low in neuroticism and extraversion/openness vs. conscientiousness/agreeableness in the accounts’ distribution by their personality traits were identified. Conscientiousness was the trait most associated with photo features—in particular, with content category, pixel-color, and visual features, while agreeableness was the trait least associated with photo features. Neuroticism was mainly correlated with pixel-level features, openness was correlated mainly with pixel-color features, and extraversion was correlated mainly with facial features. The personality traits, except neuroticism, were predicted from the photo features.

**Conclusions:**

This study applied the theoretical lens of personality, which has been mainly used to examine individuals’ behaviors, to investigate the SNS communication of startups. Moreover, it focused on the visual communication of organizational accounts, which has not been actively studied in the literature. This study has implications for expanding the realm of personality research to organizational SNS accounts.

## Introduction

Personality has been examined in terms of its relationship with individuals’ use of social networking sites (SNSs). It can be defined as consistent patterns of thought, emotion, and behavior across time and situations, and it can identify the aspects how individuals differ among many possible dimensions of difference [1]. Thus, individuals with different personality may use SNSs in different ways. In particular, as content producers rather than consumers, individuals may upload posts with different characteristics according to their personality [2]. In the literature, this relationship between personality and posts’ characteristics has been mainly investigated in two ways. SNS users’ personality was assessed based on personality models such as the Big Five, and the posts’ differences were determined [3]; alternatively, the features of SNS posts were extracted to be used for predicting the uploaders’ personality [4].

This investigation can be applied to examining organizational SNS accounts as well as individual ones. Organizational and individual accounts are similar in terms of their online behaviors: they can upload posts, become friends or follow other accounts, and comment or like others’ posts. That is, organizational accounts present themselves in the same way as personal accounts [5]. Thus, as individual users’ personality is reflected on and predicted from the posts they upload, the personality of organizational accounts can be examined in terms of its relationship with the posts’ characteristics. This application of personality to organizational SNS accounts aligns with the expansion of personality to nonhuman objects in the literature. Given that personality is assessed based on the manifested differences in behaviors, it can be applied to various nonhuman species and inanimate objects where each individual unit has distinct behavioral markers from other units. Accordingly, not only animals but also products, stores, brands, and websites have been investigated from the perspective of personality [6], and organizational SNS accounts can be another domain of its application.

Among various types of organizations, this study focuses on startups, which can be generally defined as new and small to medium-sized enterprises that encourage novel ideas and seek business opportunities based on innovative technologies [7]. As startups usually lack enough monetary or human resources to perform large-scale advertising on mass media or public relation campaigns, they can make use of SNSs as a viable alternative. Further, SNSs enable startups to communicate with many stakeholders, such as investors, cooperators, customers, and employees, and they have been considered critical for startups’ outcome [8]. In this regard, many studies have shown that startups’ active SNS use was closely related to their performances in marketing [9], funding [10], and even survival [11]. However, while few studies have analyzed the content of tweets uploaded by startups’ founders or CEOs [7, 12], scant research has focused on the characteristics of posts uploaded to the startups’ SNS accounts.

Meanwhile, this study examines photos uploaded to Instagram accounts. Previous studies on personality and SNS posts have mainly analyzed text data [13], which remains the dominant format of posts. However, the share and importance of visual posts have been rapidly growing on SNSs. Users of extant SNSs such as Facebook and Twitter upload posts with photos, and photo-centric SNSs such as Instagram have also become popular. In this context, previous studies have investigated how the characteristics of SNS photos are related to the uploaders’ personality [3]; however, they mainly focused on the photos of individual users, largely overlooking organizational SNS accounts. In addition, previous studies have paid limited attention to the photos’ characteristics at the pixel-level, where information is delivered and meaning is created as been done at the content-level.

Based on the above considerations, this study aims to examine the personality of startups’ Instagram accounts and investigate its relationships with the uploaded photos’ characteristics at the content and pixel levels. To this aim, Big Five personality traits of the accounts are assessed using an online artificial intelligence (AI) service, which has been used in previous studies and is known to be equally or more accurate than human assessment [14]. Moreover, photo features at the content and pixel levels are extracted to examine how they are associated with the personality traits and whether they can predict the traits.

The remainder of this paper is structured as follows. Previous studies on the personality of nonhuman objects, startups’ SNS posts, and the relationship between personality and SNS posts are reviewed. Then, it is described how the research sample was selected, how the personality traits of startups’ accounts were measured, and which photo features were used for analysis. Finally, the results of the analyses are presented, the implications and limitations of this research are discussed, and topics for further research are suggested.

## Theoretical backgrounds and related works

The theoretical backgrounds and the key concerns in related research questions are summarized in Table 1 below for readability. Each part is introduced in each of following subsections.

**Table 1:** Summary of Theoretical Backgrounds (Related References) and Key Concerns in Research Questions

Overall	Specific	Key Concern in RQ
The Big-Five Personality model has been applied to nonhuman objects ( [1, 6, 15–22]).	Startups’ SNS posts have been analyzed ( [7–11, 23, 24]) but not from the perspective of personality.	RQ1. Characteristics of the personality of startups’ Instagram accounts
	SNS users’ personality was found to be related to the characteristics of their posts ( [2, 3, 13, 26–28]).	RQ2. Relationships between the personality of startups’ Instagram accounts and their photo features
	SNS photo features has been used to predict uploaders’ personality ( [4, 29–34]).	RQ3. Accuracy of predicting the personality of startups’ Instagram accounts from their photo features

### Big five personality model and personality of nonhuman objects

One of the most widely used models in analyzing human personality is the Big Five [1], which represents human personality by following five traits and their relative strengths. Openness is the propensity for being intellectually curious, having broad interests, and seeking novelty—a trait also related to being imaginative and artistic. Conscientiousness is characterized by being responsible, reliable, careful, and hard-working; individuals with high conscientiousness are known to lead their lives in disciplined and well-organized manners. Extraversion reflects the disposition to be outgoing, gregarious, and socially active; highly extroverted individuals usually enjoy interaction with others and do not mind to be the center of attention. Agreeableness refers to the tendency to be generous, altruistic, and compliant with others—a trait also related to the ability to sympathize with and consider for others. Finally, neuroticism—also known as emotional instability—characterizes individuals who are anxious, temperamental, and depressed; highly neurotic individuals usually have negative emotions about themselves and their surroundings.

The Big Five model has been adopted to examine the personality of nonhuman objects. Since personality is the pattern of difference in behaviors among individuals, external judges can observe and classify an object’s behaviors and appearances to assess its personality. In this regard, owners of or experts on animals, such as dogs, horses, or chimpanzees, have assessed their personalities [15]. The same approach has been applied to inanimate objects. The personalities of products [6] and stores [16] have been assessed and found to be related to the products’ popularity [17] and the store loyalty [18]. Moreover, websites’ personality [19] has been investigated and found to be associated with users’ purchase intention [20] and satisfaction [21].

However, the Big Five model has been applied limitedly to examining the organizational SNS accounts, whose online behaviors are similar to those of personal accounts. According to the Computers Are Social Actors paradigm [22], people apply human social rules when they interact with computers because they perceive computers as human beings. In a similar vein, SNS users would apply the same social rule when interacting with organizational accounts, and they would perceive the personality of organizational accounts as they would that of individual accounts. Posts on accounts are the sites where personality is (expressed and) perceived; thus, they can be the major target of analysis to assess the personality of SNS accounts—both individual and organizational.

### Analysis of startups’ SNS posts

Some studies have examined the topics or communication strategies manifested in the SNS posts of startups. These studies have analyzed the accounts of startup founders or CEOs rather than organizational accounts. For example, Sindhani et al. [12] identified key topics in startup CEOs’ tweets, such as business situations, personal feelings, and societal concerns. Yue et al. [7] conducted a content analysis of startup CEOs’ tweets to show their communication strategies, and their results suggested that startup CEOs did not fully utilize the dialogic principle in Twitter communication.

Other studies have examined the relationship between startups’ SNS use and their performances. Jung and Jeong [9] analyzed the tweets of startup firms and showed the significant relationships between the activity level, measured by number of tweets, retweets, and likes, and SNS users’ engagement. Wang et al. [23] reported the associations between the informativity, persuasiveness, and transformity of the startup founders’ tweets and their engagement. Other studies have found that startups’ active SNS use was directly related to their business performances: the activity levels on Facebook [24], Twitter [10], and LinkedIn [8] were related to the funding outcomes. Further, startups’ active SNS use was one of the predictors of the company’s 5-year survival; specifically, the number of likes on Twitter was the most important digital trace predicting the company’s survival [11].

However, startups’ SNS posts have not been examined from the perspective of personality. While few studies have evaluated the personality of the SNS accounts of brands [5] and accommodation platforms [25], those of startups have not been analyzed. This study tries to fill this gap by examining the personality of startups’ Instagram accounts. The following research question is raised:

RQ1. What are the characteristics of the personality of startups’ Instagram accounts?

### Personality traits and the characteristics of SNS posts

SNS users’ personality has been investigated in terms of its relationship with the characteristics of their posts, and their content has been the major target of investigations. For example, Winter et al. [26] showed that Facebook users with a high level of extraversion uploaded posts about themselves more frequently and in more detail. Pentina and Zhang [27] examined how the emotions expressed on Facebook posts differed by the uploaders’ personality, and their results suggested that extraversion, agreeableness, and conscientiousness were significantly associated with disclosing positive emotions. Miller [13] showed that Facebook users with a high level of conscientiousness uploaded less on inappropriate material such as alcohol, drug, gun, and sexist and racial comments.

It has been found that the characteristics of photos, as well as texts, on SNS are related to the uploaders’ personality. Wu et al. [2] showed significant relationships between the characteristics of Facebook profile photos and the uploaders’ Big Five personality traits, among which extraversion showed the strongest association. Guntuku et al. [28] reported that more open users were more likely to post artistic photos, and more conscientious users were more likely to post building- and office-related photos on Twitter. Saitov et al. [3] analyzed the photos on VK, a Russian SNS, and reported significant associations between high conscientiousness and face photos, low extraversion and cat photos, and low agreeableness and landscape photos.

The literature has thus shown that the characteristics of SNS posts are related to the uploaders’ personality; however, this approach has hardly been applied to analyzing posts on organizational SNS accounts. The similarity between personal and organizational accounts in terms of their online behavior can lead to presume that the relationship between personality and the characteristics of SNS posts also holds in organizational accounts. Based on this consideration, the following research question is proposed:

RQ2. How is the personality of startups’ Instagram accounts related to the characteristics of their photos?

### Predicting uploaders’ personality from photo features

SNS data can be considered as digital footprints that have been used to predict behavioral and psychological characteristics, including the Big Five personality traits [29]. They have drawn attention as a non-intrusive measurement technique because the computer-based prediction of traits using SNS data is known to be equally of more accurate than human prediction [14]. In particular, photo data have been reported as more accurate than text data in predicting uploaders’ personality [29, 30].

Previous studies have used various kinds of SNS photos to predict uploaders’ personality, such as profile photos [4], selfies [31], liked photos [32], and all photos [33]. Meanwhile, various kinds of features extracted from SNS photos have been used to predict the uploaders’ personality. On the one hand, features regarding photos’ characteristics, such as content and color, have been manually extracted based on particular algorithms [33, 34]; on the other hand, the parameter values in a particular layer of a deep learning model have been used as predictive features [31]. While the features based on deep learning models can have better predictive accuracy, manually extracted features can be more useful for humans to understand their meanings.

In line with the abovementioned studies, this research predicts the personality of startups’ Instagram accounts using the features extracted from their photos. Given the similarities in online behaviors between individual and organizational SNS accounts, it can be presumed that the personality of organizational accounts can be predicted as has been done in individual accounts. The following research question is proposed:

RQ3. How is the personality of startups’ Instagram accounts predicted from the characteristics of their photos?

## Methods

### Research sample

The list of startups was obtained from the Best Startup Companies page [35] provided by Fundz, a startup database, and the author visited the official webpage of each company to obtain its Instagram account. Organizations without Instagram accounts or with inactive accounts showing less than 30 posts were excluded from the research sample. As a result, 108 Instagram accounts of startups were selected (Table 2).

The data were collected using Instagram-Scraper-2021 [36]; this basically gathers data from the HTTP Archive (HAR) file, which stores the tracking information about the interaction between the web browser and the website. For each account, the program opened the account’s Instagram profile page, scrolled by page three times, and saved the HAR file. The profile page and each scrolled page displayed about 15 posts; thus, the aim was to obtain about 60 recent posts. However, since some accounts had fewer posts, 56.73 posts on average (SD = 5.87) per accounts were collected. From the HAR file, the photo part, which was transformed from Base64 into JPG format, and the metadata part, which was saved in JSON format, containing caption texts for each post were used for analysis.

**Table 2:** Instagram Accounts of Startups in the Research Sample

accounts	
305fitness, 98point6, acloudguru, airtable, allbirds, altopharmacy, alycegifts, attentivemobile, weareauth0, babylonhealth, bellhopmoving, bestow_life, betterdotcom, bloomscape, branchmetrics, brexhq, brooklinen, livebungalow, calendlyhq, calm, cameo, capsulecares, gocapway, carbonhealth, checkrinc, cockroachdb, confluent_inc, curativeinc, curology, dailyharvest, dandelion, databricksinc, dataiku, thedaveapp, dipseastories, divvyhomes, deliverrinc, doordash, drift, duolingo, wearedutchie, emergeculture, envoy.inc, fabfitfun, faire_wholesale, fig.agency, figuretechnologies, flatironhealth, forward, fronthq, fullstoryhq, grovecollaborative, guildeducation, hacker0 × 01, harness.io, havenlifeinsurance, hawkemedia, headspace, healthsherpas, hims, hiyainc, homebound, hover3d, impossible_foods, uselatticehq, leadgenius, lead_iq, lemonade_inc, localize_app, masterclass, mirohq, modernhealthco, mural_app, newfrontinsurance, notionhq, novacredit, ntwrk, odettainc, outreach.io, pvolve, patient_pop, petalcard, policygenius, poshmark, productboard, ridgeline_apps, ripplinghr, robinhoodapp, lifeatsamsara, sanabenefits, seamless.ai, sendosohq, shieldaicareers, spatial_io, superhumanco, talkdesk, the.wing, trellahealth, trustedhealth, tula, turingcom, uniteushq, unqork, hello.upkey, verkadahq, wellhealthinc, getwrench, zoom	

### Measuring the personality of organizational accounts

The Big Five personality traits of each account were measured using IBM Watson Personality Insights. When users upload texts to the server, the pretrained AI model infers the personality of their writer based on previous research on the relationship between language and personality [37]. This service has been used in the literature to assess the personality of writers of various texts [38] including SNS posts [39]. In this study, the caption texts of all posts uploaded to an account were sent to the server via application programming interface (API), and the service returned the account’s Big Five personality traits—openness, conscientiousness, extraversion, agreeableness, and neuroticism—by a value between 0 and 1 for each trait.

### Photo features

Photo features were extracted based on previous studies about the personality of SNS users and the characteristics of their photos [30, 32, 40]. The features were extracted at the content (content category and facial features) and pixel (pixel color and visual features) levels. As the unit of analysis in this study is the account, the features extracted from each photo were averaged across all photos of a given account except for the content category features which were already account-level metrics.

#### Content category features

Each photo was classified into one of the given categories based on its content by Computer Vision API in Microsoft Azure Cognitive Services. Each photo was sent to the server via API, and the pretrained AI categorized the photos’ content into one of the 15 predetermined classes: *abstract*, *animal*, *building*, *dark*, *drink*, *food*, *indoor*, *others*, *outdoor*, *people*, *plant*, *object*, *sky*, *text*, and *transportation*. And the share of photos in each content category was measured. For example, if the *animal* of an account was 0.5, half of the photos uploaded to the account were of animal. Additionally, the *Gini* coefficient was measured as a metric of non-diversity (concentration) of the photos in terms of their content.

#### Facial features

The features of human faces in photos were extracted using Face API in Microsoft Azure Cognitive Services. For a given photo sent to the server, the pretrained AI detected human faces in the photo and returned the estimated attributes of each face, including age, gender, and emotions. The returned results were used to extract the following features. The *number of faces* accounted for how many faces appeared in a photo, *closeup* represented the ratio of the size of the biggest face in a photo to the photos’ total size; and *face ratio* represented the ratio of the sum of sizes of all faces in a photo to the photos’ total size. *Age* referred to the average age of faces, and *gender* captured the number of female faces in a photo. Moreover, the facial emotions on all faces in a photo were averaged by each emotion (*anger*, *contempt*, *disgust*, *fear*, *happiness*, *sadness*, *surprise*, and *neutral*), which was assessed as a real number between 0 and 1.

#### Pixel color features

Pixels in digital photos contain information about colors, which are expressed by color space models such as RGB (red, green, blue) and HSV (hue, saturation, value). This pixel color information was used to extract the following features in the Python programming language and the OpenCV library.

The means and variances of RGB, saturation, and value (i.e., lightness) were calculated respectively across all pixels in a photo: the resulting features were *red mean*, *red variance*, *green mean*, *green variance*, *blue mean*, *blue variance*, *saturation mean*, *saturation variance*, *value mean*, and *value variance*. Since hue is nominal, unlike saturation and value, the number of peaks in hue histogram (*hue peaks*) was extracted, rather than mean and variance. A hue histogram was generated and smoothed by kernel density estimation, and the number of local maxima was counted: this metric was reported to represent a photo’s degree of being monotonous or mussy [41].

#### Visual features

The features concerning a photos’ visual attractiveness were extracted based on previous studies [42, 43]. *Brightness* refers to how bright a photo is and was measured by the average of luminance in the photos’ pixels. *Colorfulness* represents how colorful a photo is and was measured by the means and standard deviations of metrics composed of relative amounts of RGB values in the pixels. *Naturalness* accounts for how much a photo corresponds to the human perception of reality and was measured using the proportion of pixels whose saturation and luminance fall within a certain range. *Contrast* captures the relation of local luminance variations to the surrounding luminance and was measured by the standard deviation of luminance in pixels divided by the number of pixels. In addition, *RGB contrast* was measured by extending contrast into the three-dimensional RGB color space. *Sharpness* refers to a photo’s clarity and level of detail and was measured by a function of Laplacian of each pixel’s luminance, normalized by the local average luminance in the surroundings of each pixel. Two additional visual features concerning color were measured. *Color diversity* represents how diverse the colors in a photo are and was measured by the fractal dimension using the box-counting method [44]. *Color harmony* accounts for how harmonious the dominant colors in a photo are and was measured by the geometric formulations generated by the dominant colors on the color wheel: the two colors that correspond to the first and the second highest peaks in the smoothed hue histogram were located on the color wheel, and the internal angle between the two colors was identified as color harmony [45].

### Analytical strategies

For RQ1, the means of the Big Five personality traits of startups’ Instagram accounts were obtained, and their characteristics were examined. In addition, a correspondence analysis (CA) was conducted to identify the key dimensions where startups’ accounts were distributed by their personality. CA is a graphical data analysis method to represent a set of data points in a low-dimensional space, where the distances between the points reflect the similarity or dissimilarity between the points, and the dimensions show the key criteria of the similarity or dissimilarity [46]. For RQ2, correlational analyses were conducted between photo features and personality traits using Spearman correlation coefficients. For RQ3, predictive analyses were conducted through random forest regression models; these were built using 100 decision trees, each of which used all samples and all features. The models were trained with 10-fold cross validation, and root mean squared errors (RMSEs) were calculated. The RMSEs were compared with those in previous studies which predicted SNS user personality using photo features. For comparison, the RMSEs of previous studies measuring user personality using a 5-point Likert scale were divided by 4, the range of the 5-point scale, to convert it into the [0, 1] scale used in this study.

## Results

### Characteristics of the personality traits of startups’ Instagram accounts (RQ1)

**Fig. 1:**
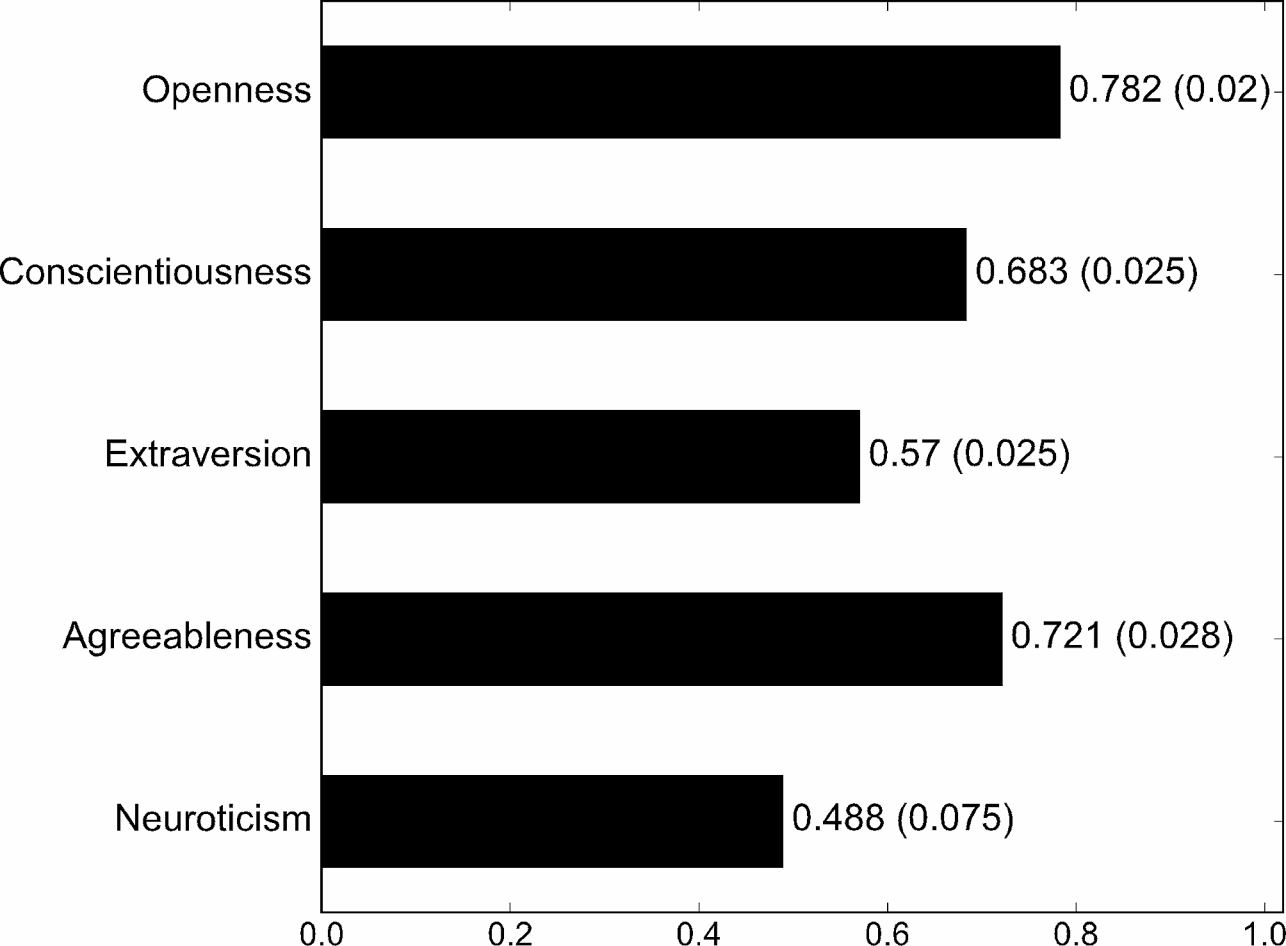
Mean (and Standard Deviation) Personality Traits of Startups’ Instagram Accounts

Figure 1 shows the mean personality traits of startups’ Instagram accounts. It indicates that the personality of startups’ Instagram accounts can be summarized as high in openness, agreeableness, and conscientiousness and middle in extraversion and neuroticism. Precisely, openness was the most dominant trait, followed by agreeableness and conscientiousness. This pattern in personality traits differs from the ones found in previous studies that analyzed the personality of individuals or organizations, such as CEOs of S&P 500 companies [38], software developers [39], and hotels and accommodation platforms’ Twitter accounts [25], using IBM Watson Personality Insights.

**Fig. 2:**
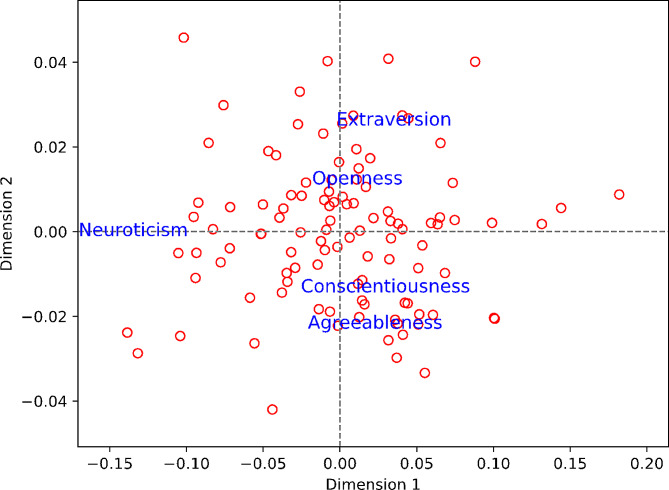
Correspondence Analysis Plot of the Personality of Startups’ Instagram Accounts. Each Circle Stands for Each Instagram Account Based on its Personality Traits

Next, Fig. 2 presents the result of CA. It shows that the first dimension (horizontal axis) was about being high vs. low in neuroticism, and the second dimension (vertical axis) was about being high in extraversion and openness vs. high in conscientiousness and agreeableness. Startups’ accounts are distributed quite evenly along the two dimensions, which suggests that the two dimensions are the key axes to discern the characteristics of personality traits of startups’ Instagram accounts.

### Correlations between personality traits of startups’ Instagram accounts and their photo features (RQ2)

Table 3 presents the results of the correlational analyses between personality traits and photo features; they are discussed in the following subsections.

**Table 3:** Spearman Correlations Between the Personality Traits of Startups’ Instagram Accounts and the Features of Their Photos

	Feature	Openness	Conscientiousness	Extraversion	Agreeableness	Neuroticism
Content category	Abstract	0.237*	− 0.016	− 0.171	− 0.005	0.192*
	Animal	− 0.020	0.255*	0.132	0.011	0.035
	Building	0.082	− 0.036	− 0.106	− 0.130	− 0.172
	Dark	− 0.007	− 0.086	− 0.260*	0.147	0.103
	Drink	0.218*	0.226*	0.102	0.046	0.070
	Food	0.150	0.414*	0.113	0.133	− 0.150
	Indoor	0.094	0.390*	0.119	0.286*	− 0.150
	Others	0.014	− 0.110	− 0.239*	− 0.043	0.124
	Outdoor	0.008	0.075	− 0.043	0.122	− 0.194*
	People	− 0.097	0.380*	0.181	0.398*	− 0.031
	Plant	0.172	0.079	0.033	0.008	− 0.070
	Object	− 0.015	0.057	0.006	0.005	0.018
	Sky	0.202*	− 0.051	0.063	0.040	0.183
	Text	− 0.014	− 0.494*	− 0.091	− 0.366*	0.024
	Transportation	0.159	− 0.008	0.016	− 0.086	− 0.066
	Gini	− 0.230*	− 0.248*	0.098	− 0.134	− 0.002
Facial features	Number of faces	− 0.164	− 0.067	0.240*	0.080	0.023
	Face ratio	− 0.086	− 0.106	0.218*	− 0.032	0.024
	Closeup	− 0.065	− 0.130	0.206*	− 0.061	0.025
	Age	− 0.102	− 0.139	0.233*	0.021	0.048
	Gender	− 0.015	0.041	0.369*	0.176	− 0.002
	Anger	− 0.176	0.091	0.054	0.008	− 0.062
	Contempt	− 0.148	− 0.067	− 0.069	− 0.117	− 0.002
	Disgust	− 0.040	0.108	0.045	0.021	− 0.059
	Fear	− 0.151	0.047	0.067	− 0.032	− 0.038
	Happiness	− 0.131	− 0.055	0.297*	0.077	− 0.010
	Sadness	− 0.109	0.055	0.104	0.115	0.031
	Surprise	− 0.227*	− 0.072	0.091	− 0.064	− 0.107
	Neutral	− 0.072	− 0.152	− 0.012	− 0.070	0.142
Pixel color features	Red mean	0.235*	− 0.103	0.021	− 0.144	0.190*
	Red variance	− 0.250*	0.167	0.093	0.115	− 0.262*
	Green mean	0.178	− 0.239*	− 0.010	− 0.191*	0.172
	Green variance	− 0.275*	0.177	0.053	0.072	− 0.238*
	Blue mean	0.179	− 0.313*	− 0.021	− 0.174	0.248*
	Blue variance	− 0.189	0.212*	− 0.065	0.052	− 0.241*
	Saturation mean	− 0.099	0.142	0.113	0.202*	− 0.120
	Saturation variance	− 0.104	0.188	0.059	0.031	− 0.105
	Value mean	0.217*	− 0.202*	0.036	− 0.128	0.191*
	Value variance	− 0.296*	0.213*	0.059	0.082	− 0.275*
	Hue peaks	− 0.089	− 0.295*	− 0.131	− 0.235*	− 0.046
Visual features	Brightness	0.212*	− 0.224*	− 0.002	− 0.177	0.213*
	Colorfulness	− 0.064	0.090	0.103	0.142	− 0.086
	Naturalness	− 0.107	0.320*	0.070	0.020	− 0.176
	Contrast	− 0.220*	0.233*	0.041	0.084	− 0.221*
	RGB contrast	− 0.216*	0.229*	0.043	0.080	− 0.260*
	Sharpness	− 0.104	0.235*	0.100	0.052	− 0.203*
	Color diversity	0.010	0.555*	0.265*	0.337*	− 0.148
	Color harmony	− 0.124	0.127	0.173	0.179	− 0.201*

Note. *: *p* <.05

#### Correlations between openness and photo features

Concerning content-level features, the negative correlation between openness and Gini suggests that more open accounts had more diverse photos in terms of content—a result consistent with the meaning of openness, namely having a wide range of interests and seeking new experiences [1]. A similar result was found in previous studies that analyzed photos in individual accounts [47]; therefore, it can be argued that this aspect is shared by both individual and startups’ accounts. Additionally, openness was associated with the share of photos of abstract, drink, and sky. Furthermore, openness was hardly correlated with facial features, and only surprise was negatively correlated as reported in the literature [40].

Concerning pixel-level features, openness was found to be correlated with value mean and brightness; in other words, the photos of more open accounts were brighter. These results contradict those of previous studies that analyzed photos in individual accounts [28], namely that the photos of more open users were less bright. Such results can be ascribed to the other aspect of openness—that of being artistic and imaginative [1]; more open users might upload photos with more artistic attempts, such as extreme close-ups or gray-scaled photos, which usually lower brightness [40]. However, this was not the case in startups’ Instagram accounts: the artistic aspect of openness was not manifested. The same can be said for the correlations between openness and (RGB) contrast: openness was associated with them positively in individual accounts in previous studies [40, 48] but negatively in startups’ accounts in this study. Additionally, openness was associated positively with red mean and negatively with red and green variances.

#### Correlations between conscientiousness and photo features

The correlations between conscientiousness and content category features found in this study show two aspects: the one that is consistent with the trait’s meaning and the results in previous studies, and the one that is not. On the one hand, conscientiousness was correlated with the share of photos of indoor and people. Given that conscientiousness is linked to being responsible, reliable, disciplined, and well-organized [1], these results are understandable and the same results have been reported in previous studies [3, 28]. On the other hand, however, conscientiousness was negatively associated with the share of photos of text. The positive association between conscientiousness and the share or building- and office-related photos, reported in Guntuku et al. [28], may lead to presume that texts would appear considerably in the photos uploaded to conscientious accounts. Nevertheless, our result suggests the opposite, namely that more conscientious accounts uploaded less text-related photos. Additionally, other significant correlations are incommensurate with the trait’s meaning: conscientiousness was positively associated with the share of photos of animal, food, and drink, which are usually inharmonious with being hard-working [1].

Concerning pixel-level features, the photos of more conscientious accounts were less bright: conscientiousness was negatively correlated with value mean and brightness. This was also the case with the means of blue, green, and red (while insignificant), suggesting that the photos of more conscientious accounts contained a lesser amount of color. These are inconsistent with the literature that analyzed individual accounts, in which more conscientious users uploaded brighter photos [40, 48]. In contrast, some features were associated with conscientiousness in the same way they were in the literature that analyzed individual accounts [28, 40, 48]: the photos of more conscientious accounts were more natural, more detailed (sharper), more diverse in color, and had more contrast. In sum, the conscientiousness of startups’ Instagram accounts manifested two aspects of correlations with pixel-level features: the one that is the same in individual accounts in the literature, and the other that is characteristic to startups’ accounts.

#### Correlations between extraversion and photo features

Based on the meaning of extraversion, it can be presumed that more extroverted accounts might have a higher share of photos about their social activities—photos of people, outdoor, drink, or food. None of them, however, was significantly associated with startups’ extraversion. Instead, the trait was more correlated with facial features; more extroverted accounts had more and larger faces in their Instagram photos (correlated with the number of faces, face ratio, and closeup). In addition, the faces in the photos of more extroverted accounts exhibited happier emotion. These results are consistent with the meaning of extraversion and the literature that analyzed individual accounts [34, 40, 48, 49]. However, other results were in contrast with those in the literature: more extroverted accounts had older (correlated with age) and more female (correlated with gender) faces in their photos, which can be said to be a characteristic of startups’ accounts rather than individual ones [40].

Extraversion was hardly associated with pixel-level features as the only significant correlation was found with color diversity. While a similar result cannot be found in the literature, it can be interpreted that the active social relationships of more extroverted accounts might be manifested by more diverse colors in their photos.

#### Correlations between agreeableness and photo features

Since agreeableness is related to being generous to and sympathetic toward others [1], more agreeable accounts are expected to upload more photos of people. The positive correlation between agreeableness and share of photos of people is consistent with the expectation, and it also corresponds with the results in the literature that analyzed the photos in individual accounts [3, 28]. Additionally, agreeableness was negatively associated with the share of photos of text: this result was hardly found in the literature and can be a characteristic of startups’ accounts. Finally, none of the facial features were correlated with agreeableness.

Concerning pixel-level features, saturation mean and color diversity were associated positively with agreeableness, meaning that, consistent with the literature [28], the photos of more agreeable accounts were stronger and more diverse in color. Moreover, the negative correlation between agreeableness and hue peaks suggests that the photos of more agreeable accounts were more monotonous.

#### Correlations between neuroticism and photo features

Neuroticism was negatively correlated with the share of photos of outdoor scenes. While this result was hardly found in the literature, it can be interpreted that the anxious and depressed disposition of neurotic individuals [1] would lead them to perform less outdoor activities and upload less photos of them. This was also the case in startups’ Instagram accounts in this study. Concerning facial features, the literature has reported associations between neuroticism and negative emotions manifested in individual accounts’ SNS posts [40]. This, however, was not the case in startups’ Instagram accounts in this study, where no facial feature was significantly correlated with neuroticism.

Concerning pixel-level features, the photos on more neurotic accounts were brighter, that is, value mean and brightness were correlated with neuroticism. However, they were less diverse in brightness as neuroticism was negatively correlated with value variance. This pattern—positive correlation with mean and negative correlation with variance—was also found between neuroticism and RGB colors (while it was insignificant with green mean): the photos in more neurotic accounts were larger in means of RGB but smaller in their variances. Based on the meaning of neuroticism, it can be presumed that the depressed and instable emotion of neurotic people would make their photos weaker and inconstant in color and brightness. The result of this study, however, revealed the opposite, which can be considered characteristic of startups’ accounts. In contrast, other results are consistent with the meaning of neuroticism. For example, neuroticism was negatively correlated with sharpness, color harmony, and (RGB) contrast: the photos in more neurotic accounts were more blurred, less harmonious in color, and had low (RGB) contrast. The relationship between neuroticism and pixel-level characteristics has rarely been reported in the literature, and our results are expected to contribute to it.

### Predicting personality traits using photo features (RQ3)

Table 4 presents the RMSEs of random forest regression models that predicted the personality traits of startups’ Instagram accounts from their photo features. RMSEs were between 0.125 and 0.240 when all features were used for prediction, and they were compared with the ones in previous studies: they were 0.127–0.22 [50], 0.165–0.252 [30], 0.188–0.219 [31], and 0.202–0.278 [32]. The comparison suggests that the Big Five personality traits of startups’ Instagram accounts can be predicted from the features of their photos with an acceptable level of accuracy. It should be noted, however, that the RMSE of neuroticism was the largest: the prediction of neuroticism was the least accurate. This result is consistent with previous studies that reported neuroticism as the most difficult personality trait to predict [49].

**Table 4:** Root Mean Square Errors in 10-fold Cross Validation of Random Forest Regression on Personality Traits

Feature	Openness	Conscientiousness	Extraversion	Agreeableness	Neuroticism
Content category	0.121	0.130	0.145	0.145	0.241
Facial	0.130	0.140	0.139	0.150	0.241
Pixel color	0.128	0.138	0.146	0.147	0.252
Visual	0.127	0.123	0.147	0.147	0.243
All	0.125	0.124	0.140	0.139	0.240

## Discussion

The major findings of this study can be summarized as follows. The personality of startups’ Instagram accounts was high in openness, agreeableness, and conscientiousness and middle in extraversion and neuroticism. This pattern in Big Five personality traits is distinct from those of other individuals or organizations whose personality was assessed from their SNS posts. In addition, the personality of startups’ Instagram accounts was distributed along two dimensions of high vs. low in neuroticism and extraversion/openness vs. conscientiousness/agreeableness, which show how personality is different among the accounts. Personality traits were associated with a part of photo features. Conscientiousness was the trait most associated with photo features—in particular, with content category, pixel-color, and visual features. Neuroticism was mainly correlated with pixel-level features, openness was correlated mainly with pixel-color features, and extraversion was correlated mainly with facial features. In contrast, agreeableness was the trait least associated with photo features. Finally, the personality traits of startups’ Instagram accounts—except neuroticism—were predicted from their photo features.

This study has implications for expanding the realm of personality research. In the literature, personality has been applied to understand the behaviors of various nonhuman objects [15]; however, this type of research has not actively focused on organizational SNS accounts. This research fills this gap, showing that startups’ Instagram accounts have a distinct pattern in personality traits and that the characteristics of their posts are related to their personality. This study is also meaningful for the study of self-presentation in visual forms. The literature has reported that personality is related to self-presentation on SNSs [51]. This study contributes to this line of research by showing that visual self-presentation is associated with and can be a predictor of personality. In particular, the pixel-level characteristics, as well as the content-level ones, of visual self-presentation are related to personality. Further, this study can have implications for visual communication research. More and more people are using visual mode of communication, and it is important to understand how individuals and organizations use it for their everyday and professional purposes [52]. The present research can contribute to the understanding by investigating the relationship between the characteristics of social media users and the features of the photos they upload. Finally, this study has implications in the context of organizational communications. While few previous studies have analyzed the personality traits of particular organizations [5, 25], they have not investigated how such traits are related to their online behaviors, such as uploading posts. This study not only identified the pattern in personality traits of startups’ Instagram accounts but also showed that personality is related to the characteristics of their photos, which reflect their online behaviors. These findings would contribute to the understanding of how organizations’ online behaviors would differ by their accounts’ personality. In terms of the type of photos used for analysis, this study can be meaningful as it showed that the personality can be predicted accurately from some recent photos, as done using all [30, 33], profile [4], selfie [31], or liked photos [32] in the literature.

This research also has practical implications. Based on this research, organizations can examine and improve how they are presented online to the public. For SNS users, interacting with an organizational account with a certain personality (e.g., high in openness and low in extraversion) might feel like interacting with an account of an individual with the same personality. Thus, organizations can manage their online impression so that the public might feel in a certain way from interacting with their accounts. The literature has suggested that the personality of followers of organizational accounts corresponds with that of the accounts themselves [5]; thus, it can be presumed that posts that are more consistent with the personality of organizational accounts would appeal more to their followers. The results of this study can contribute to increase the appeal. For example, more conscientious accounts need to design their photos to show more of indoor and people and less of text, and more extroverted accounts need to make their photos to reveal happier emotions on faces and exhibit more diverse colors.

The main limitation of this study is that the research sample was obtained from a single source (Fundz) thus the generalizability of the results can be limited. Future research may include startups from larger and more diverse sources in the research sample in order for the results to represent all startups companies. And the exclusion of inactive accounts from the research sample could introduce a bias. In this study, the accounts with less than 30 posts were excluded, but this could lead to the more exclusion of the business-to-business companies than the business-to-consumer companies because the former could be more inactive in SNS than the latter. Another limitation can be potential biases associated with automated text analysis tools. This study used IBM Watson Personality Insights to assess the personality traits of social media accounts from their posts, but this tool may overlook the aspects of traits that cannot be detected by texts and this may generate biases in assessing personality traits. Also, this study did not examine how the personality of startups’ accounts differs by sectors. Accounts of startups in different sectors such as technology, fashion, or health can have different personality, and they might show differences in the relationships between personality and photo characteristics. Moreover, this research did not investigate whether and how the personality startups’ accounts changed according to their growth; startups can have different personality when they have just started, when they have succeeded in receiving fundings, or when they have released new products or services. Future research with a larger and more diverse sample is expected to address these issues. Additionally, future research can examine how the characteristics of startups’ Instagram photos are related to their performances, such as engagement and business profit, and how the relations differ by their personality.

## Conclusions

This study applied the theoretical lens of personality, which has been mainly used to examine individuals’ behaviors, to investigate the SNS communication of startups. Moreover, it focused on the visual communication of organizational accounts, which has not been actively studied in the literature. It identified the characteristics of personality traits of startups’ Instagram accounts and reveal the relationships between the traits and the features extracted from their photos at the content and pixel levels.

## Data Availability

The datasets used and/or analyzed during the current study are available from the corresponding author on reasonable request.

## Abbreviations

SNS: Social Networking Sites
HAR: HTTP Archive
API: Application Programming Interface
RGB: red, green, blue
HSV: hue, saturation, value
CA: correspondence analysis
RMSE: root mean squared error

## References

[1] McCrae RR, John OP (1992). An introduction to the five-factor model and its applications. J Pers.

[2] Wu Y-CJ, Chang W-H, Yuan C-H (2015). Do Facebook profile pictures reflect user’s personality?. Comput Hum Behav.

[3] Saitov I, Surikov A, Gorokhovatsky L (2021). Analysis of the relationship between the users personality traits and the images they post on social media. Procedia Comput Sci.

[4] Kanchana TS, Zoraida BSE (2020). Analysis of social media images to predict user personality assessment. IJEET.

[5] Yun JT, Pamuksuz U, Duff BRL (2019). Are we who we follow? Computationally analyzing human personality and brand following on Twitter. Int J Advert.

[6] Jordan PW, Green WS, Jordan PW (2002). The personality of products. Pleasure with products: beyond usability.

[7] Yue CA, Thelen P, Robinson K, Men LR (2019). How do CEOs communicate on Twitter? A comparative study between Fortune 200 companies and top startup companies. Corp Commun.

[8] Banerji D, Reimer T (2019). Startup founders and their LinkedIn connections: are well-connected entrepreneurs more successful. Comput Hum Behav.

[9] Jung SH, Jeong YJ (2020). Twitter data analytical methodology development for prediction of start-up firms’ social media marketing level. Technol Soc.

[10] Jin F, Wu A, Hitt L. Social is the new financial: How startup social media activity influences funding outcomes. Acad Manag Proc. 2017;2017:13329.

[11] Antretter T, Blohm I, Grichnik D. Predicting startup survival from digital traces: Towards a procedure for early stage investors. International Conference on Information Systems (ICIS). 2018.

[12] Sindhani M, Parameswar N, Dhir S, Ongsakul V (2019). Twitter analysis of founders of top 25 Indian startups. J Glob Bus Adv.

[13] Miller RE (2020). College students and inappropriate social media posting: is it a question of personality or the influence of friends?. Pers Individ Differ.

[14] Hinds J, Joinson A (2019). Human and computer personality prediction from digital footprints. Curr Dir Psychol Sci.

[15] Norman M, Rowden LJ, Cowlishaw G (2021). Potential applications of personality assessments to the management of non-human primates: a review of 10 years of study. PeerJ.

[16] d’Astous A, Levesque M (2003). A scale for measuring store personality. Psychol Mark.

[17] Prieto P, Briede JC, Beghelli A, Canessa E, Barra C (2020). I like it elegant: imprinting personalities into product shapes. Int J Des Creat Innov.

[18] Suh YG, Kim E, Park M-C (2019). Inter-type differences in store personality between department stores, hypermarkets, and mobile commerce. IJMC.

[19] Lal M, Katole H (2021). Website personality: a theoretical study. Psychol Edu.

[20] Jain K, Yadav D, Kapur PK, Klochkov Y, Verma AK, Singh G (2019). The role of website personality and website user engagement on individual’s purchase intention. System performance and management analytics: Asset analytics.

[21] Akrimi Y (2016). Usability, interactivity, website personality and consumers’ responses: a case of internet service provider. Int J Electron Mark Retail.

[22] Moon Y, Nass C (1996). How real are computer personalities? Psychological responses to personality types in human-computer interaction. Commun Res.

[23] Wang F, Kuruzovich J, Yingda L. Entrepreneurs’ activities on social media and venture financing. The 50th Hawaii International Conference on System Sciences. 2017.

[24] Yang S, Berger R (2017). Relation between start-ups’ online social media presence and fundraising. J Sci Technol Policy Manag.

[25] Wang X, Cheng M, Wong IA, Teah M, Lee S (2021). Big-five personality traits in P2P accommodation platforms: similar or different to hotel brands. Curr Issues Tour.

[26] Winter S, Neubaum G, Eimler SC (2014). Another brick in the Facebook wall: how personality traits relate to the content of status updates. Comput Hum Behav.

[27] Pentina I, Zhang L (2017). Effects of social support and personality on emotional disclosure on Facebook and in real life. Behav Inf Technol.

[28] Guntuku SC, Lin W, Carpenter J, Ng WK, Ungar LH, Preotiuc-Pietro D. Studying personality through the content of posted and liked images on Twitter. The 2017 ACM on Web Science Conference. 2017.

[29] Azucar D, Marengo D, Settanni M (2018). Predicting the Big 5 personality traits from digital footprints on social media: a meta-analysis. Pers Individ Differ.

[30] Ferwerda B, Schedl M, Tkalcic M, Tian Q, Sebe N, Qi G-J, Huet B, Hong R, Liu X (2016). Using Instagram picture features to predict users’ personality. Multimedia modeling.

[31] Kachur A, Osin E, Davydov D, Shutilov K, Novokshonov A (2020). Assessing the big five personality traits using real-life static facial images. Sci Rep.

[32] Huang X, Sang J, Xu C (2022). Image-based personality questionnaire design. ACM Trans Multim Comput.

[33] Samani ZR, Guntuku SC, Moghaddam ME, Preoţiuc-Pietro D, Ungar LH (2018). Cross-platform and cross-interaction study of user personality based on images on Twitter and Flickr. PLoS ONE.

[34] Segalin C, Perina A, Cristani M, Vinciarelli A (2017). The pictures we like are our image: continuous mapping of favorite pictures into self-assessed and attributed personality traits. IEEE Trans Affect Comput.

[35] Fundz. https://www.fundz.net/startup-companies-ultimate-guide. Accessed 1 Oct 2021.

[36] Ahouzi A, Instagrm. -Scraper-2021. https://github.com/aahouzi/Instagram-Scraper-2021. Accessed 7 Oct.

[37] Yarkoni T (2010). Personality in 100,000 words: a large-scale analysis of personality and word use among bloggers. J Res Pers.

[38] Mahmoudian F, Nazari JA, Gordon IM, Hrazdil K. CEO personality and language use in CSR reporting. 2021;30:338–59.

[39] Kern ML, McCarthy PX, Chakrabarty D, Rizoiu M-A (2019). Social media-predicted personality traits and values can help match people to their ideal jobs. PNAS.

[40] Liu L, Preotiuc-Pietro D, Samani ZR, Moghaddam ME, Ungar LH. Analyzing personality through social media profile picture choice. The 10th International AAAI Conference on Web and Social Media. 2016.

[41] Mao X, Chen B, Muta I (2003). Affective property of image and fractal dimension. Chaos Solitons Fractals.

[42] Datta R, Joshi D, Li J, Wang JZ, Leonardis A, Bischof H, Pinz A (2006). Studying aesthetics in photographic images using a computational approach. Computer vision: ECCV 2006.

[43] San Pedro J, Siersdorfer S. Ranking and classifying attractiveness of photos in folksonomies. The 18th International Conference on World Wide Web. 2009.

[44] Kim D, Son S-W, Jeong H (2014). Large-scale quantitative analysis of painting arts. Sci Rep.

[45] Kim JH, Kim Y (2019). Instagram user characteristics and the color of their photos: colorfulness, color diversity, and color harmony. Inf Process Manag.

[46] Greenacre M (2017). Correspondence analysis in practice.

[47] Kim Y, Kim JH (2018). Using computer vision techniques on Instagram to link users’ personalities and genders to the features of their photos: an exploratory study. Inf Process Manag.

[48] Bhatti SK, Muneer A, Lali MI, Gull M, Din SMU. Personality analysis of the USA public using Twitter profile pictures. International Conference on Information and Communication Technologies. 2017.

[49] Celli F, Bruni E, Lepri B. Automatic personality and interaction style recognition from Facebook profile pictures. The ACM International Conference on Multimedia. 2014.

[50] Skowron M, Tkalcic M, Ferwerda B, Schedl M. Fusing social media cues: Personality prediction from Twitter and Instagram. The 25th International Conference Companion on World Wide Web. 2016.

[51] Huang C (2019). Social network site use and big five personality traits: a meta-analysis. Comput Hum Behav.

[52] Russman U, Svensson J (2017). Introduction to visual communication in the age of social media: conceptual, theoretical and methodological challenges. Media Commun.

